# Neurotherapeutic effects of quercetin-loaded nanoparticles and Biochanin-A extracted from *Trifolium alexandrinum* on PI3K/Akt/GSK-3β signaling in the cerebral cortex of male diabetic rats

**DOI:** 10.1371/journal.pone.0301355

**Published:** 2024-04-29

**Authors:** Al-Sayeda Al-Sayed Newairy, Fatma Ahmad Hamaad, Mayssaa Moharm Wahby, Mamdooh Ghoneum, Heba Mohamed Abdou

**Affiliations:** 1 Faculty of Science, Department of Biochemistry, Alexandria University, Alexandria, Egypt; 2 Department of Surgery, Charles R. Drew University of Medicine and Science, Los Angeles, California, United States of America; 3 Department of Surgery, University of California Los Angeles, Los Angeles, California, United States of America; 4 Faulty of Science, Department of Zoology, Alexandria University, Alexandria, Egypt; Helwan University, EGYPT

## Abstract

Diabetes mellitus (DM) is a severe metabolic disease that can have significant consequences for cognitive health. Bioflavonoids such as *Trifolium alexandrinum* (TA), quercetin (Q), and Biochanin-A (BCA) are known to exert a wide range of pharmacological functions including antihyperglycemic activity. This study aimed to investigate the neurotherapeutic effects of quercetin-loaded nanoparticles (Q-LNP) and BCA extracted from TA against diabetes-induced cerebral cortical damage through modulation of PI3K/Akt/GSK-3β and AMPK signaling pathways. Adult male Wistar albino rats (N = 25) were randomly assigned to one of five groups: control, diabetics fed a high-fat diet (HFD) for 2 weeks and intraperitoneally (i.p.) injected with STZ (40 mg/kg), and diabetics treated with Q-LNP (50 mg/kg BW/day), BCA (10 mg/kg BW/day), or TA extract (200 mg/kg BW/day). Treatments were applied by oral gavage once daily for 35 days. Diabetic rats treated with Q-LNP, BCA, and TA extract showed improvement in cognitive performance, cortical oxidative metabolism, antioxidant parameters, and levels of glucose, insulin, triglyceride, and total cholesterol. In addition, these treatments improved neurochemical levels, including acetylcholine, dopamine, and serotonin levels as well acetylcholinesterase and monoamine oxidase activities. Furthermore, these treatments lowered proinflammatory cytokine production for TNF-α and NF-κB; downregulated the levels of IL-1β, iNOS, APP, and PPAR-γ; and attenuated the expressions of PSEN2, BACE, IR, PI3K, FOXO 1, AKT, AMPK, GSK-3β, and GFAP. The histopathological examinations of the cerebral cortical tissues confirmed the biochemical results. Overall, the present findings suggest the potential therapeutic effects of TA bioflavonoids in modulating diabetes-induced cerebral cortical damage.

## 1. Introduction

Diabetes mellitus (DM) is a severe endocrine condition caused by either complete (type-1 DM; T1DM) or relative (type-2 DM; T2DM) insulin insufficiency [1]. T2DM is more common than T1DM in clinical practices [2]; it is characterized by a decrease in insulin sensitivity in tissues and is related to insulin resistance [3]. One of the major hallmarks of insulin resistance is impaired signal transduction in the insulin/PI3K/Akt signaling pathway, leading to an increase in GSK-3β [4]. Disruption of AMPK function is also linked to insulin resistance and T2DM progression, where AMPK is a vital cellular energy sensor and a vital regulator of metabolic homeostasis [5]. Diabetes can have unfortunate consequences for cognitive health. A common consequence of diabetes is diabetic encephalopathy, a disease mediated by diabetic neurodegeneration [6]. Many downstream effects of hyperglycemia, such as oxidative stress (OS), inflammation, and the overproduction of advanced glycation end products (AGEs) are root causes of cognitive deterioration in diabetes. Studies have shown that hyperglycemia-induced neurodegeneration results in cell damage, malfunction, and finally apoptosis in the brain [7], and T2DM is associated with higher risks for depression, stroke, Alzheimer’s disease (AD), and other cognitive problems [4].

To treat diabetes, traditional medicinal plants and their derived natural compounds have become a focus of research efforts, as they have fewer adverse effects than contemporary medicine [8]. Furthermore, natural compounds can interact synergistically with other components of the same plant, amplifying or counteracting their hazardous effects [9]. Under diabetic conditions, antioxidants and anti-inflammatory drugs can supposedly prevent cognitive impairment, and AGEs, for example, can play a crucial role in the evolution of diabetes complications by linking OS and inflammation [10]. Dietary antioxidants, particularly polyphenols, are being presented as a viable way to prevent and/or cease the pathological progression of neurodegeneration and aging [9]. Bioflavonoids are secondary polyphenolic metabolites that are abundantly found in numerous fruits and vegetables [8] and are known to exert several pharmacological effects and remarkable positive impacts on human health [9].

Recent studies have demonstrated the anti-diabetic effect of TA extract [1,8]. TA is a medicinal herb that contains numerous phytoconstituents such as flavonoids and their glycosides, isoflavonoids, and terpenoid glycosides [8]. TA also belongs to the Fabaceae family; it is an Egyptian clover that was introduced into northern India and also grown in Europe, the USA, China, and Australia. Two of the most abundant flavonoids found in TA are quercetin (Q) and Biochanin-A (BCA) [1]. They have been shown to have a number of health benefits, including reducing inflammation, protecting cells from damage, and improving blood sugar control [1,8].

Q is the most prominent flavonoid in our daily diet [10], and it has been shown to have quite a range of potential effects, including antioxidant, anti-cancer, anti-viral, anti-inflammatory, antibacterial, cardioprotective, hepatoprotective, and neuroprotective activities [7]. Moreover, Q has potential therapeutic action in the prevention and treatment of neurodegenerative diseases such as AD due to its ability to cross the blood-brain barrier [10]. In addition, herbal remedies containing phytoconstituents have also been shown to slow down brain aging. BCA is a pharmacologically active phytochemical that has been identified in many plants related to the Fabaceae family [11], and it is known as a phytoestrogen with promising neuroprotective effects as it has a structure similar to natural estrogen [12]. BCA is reported to have antidiabetic effects. These effects are thought to be mediated by a number of molecular mechanisms, including activation of AMPK, reduction of ROS production, inhibition of inflammatory enzymes, and protection of pancreatic cells [13]. Q is poorly soluble in water, unstable, has low oral bioavailability, and has low permeability. Nano-formulation can help overcome the challenges of delivering Q to the body and make it more effective [1]. BCA, on the other hand, is more soluble in water and is more stable than Q. It also has higher oral bioavailability and permeability [11].

Since there is a link between neuroinflammation and cognitive impairments caused by diabetes, we thought it was of special interest to target the cerebral cortex of diabetic rats. The current study aimed to evaluate the therapeutic effects of Q-LNP, BCA, and TA extract against diabetes-induced cerebral cortical damage. Additionally, TA bioflavonoids were scrutinized for their potential therapeutic role in modulating PI3K/Akt/GSK-3β and AMPK signaling pathways. Moreover, histopathological and immunohistochemical analyses were carried out to clarify whether there were also ameliorative effects on the restoration of cellular structures.

## 2. Materials and methods

### 2.1. Preparation of *Trifolium alexandrinum* extract

TA *Linn*. (Family: Fabaceae) plants were obtained at various phases of vegetative and blooming growth from Alexandria, Egypt, at the following locations (latitude, longitude): Khorshid (31.204224, 30.037466); Izbat Al-Thalateen (31.204962, 30.028786); Apis (31.188095, 30.007219); The tenth village, Apis (31.174290, 29.969459); Village7/8 (31.122136, 29.940813); and Ezbet Al-Aqadi (30.911161, 29.537055). Safaa Abd Elsalam Mohamed, Lecturer of Plant Taxonomy and Palynology, Biological and Geological Sciences Department, Faculty of Education, Alexandria University, collected samples and taxonomically identified them based on voucher specimens. No legal permits were needed, as permissions were granted by landowners. All collected sample plants were extracted as one sample. Plants were collected from fields in the morning and immediately taken to the laboratory, where they were taxonomically identified according to morphological characters mentioned in [14]. TA aerial parts were collected, washed with distilled water, and then dried in shade. The chopped plant was processed using an electric grinder to get a fine powder. The powdered substance was steeped in 80% methanol for one week at room temperature before being filtered using a Buchner funnel with Whatman No.1 filter paper. By using a Freeze-dry system/Lyph lock 4.5 (Labconco, Kansas city, USA), the filtrate was lyophilized till dryness. Afterwards, TA dried extracts were obtained after lyophilization, and the crude extracts were refrigerated at 4°C in a marked sterile container for further examination.

### 2.2. Isolation of quercetin and biochanin A

Q and BCA were derived from TA extract and prepared as previously reported by Abdou et al. [1]. After being dried, the crude TA extract was resuspended in distilled water, and the residue was diluted with H_2_O in triplicate and extracted with ethyl acetate (EtOAc). The EtOAc fractions were then separated into 8 fractions using a silica gel (n-hexane/EtOAc/MeOH). Fractions 6 and 8 were separated and purified independently using capillary electrophoresis and silica gel column chromatography and then eluted with EtOAc/MeOH (8:1) to generate quercetin and biochanin A. The structure of the isolate was determined using HPLC.

### 2.3. Preparation of chitosan and quercetin loaded nanoparticles

The ionic gelation of chitosan with tripolyphosphate (TPP) anions was used to make chitosan nanoparticles, as described in the literature [4]. Q-LNP were produced by mixing 4 mL of TPP solution containing quercetin with 10 mL of chitosan solution. The nanoparticles were isolated from an aqueous solution containing unassociated lyophilized quercetin by ultracentrifugation at 40,000 g and 48°C for 30 minutes.

### 2.4. Physicochemical characterization of Q-LNP

Particle size (PS), polydispersity index (PDI), zeta potential (ζ), conjugation efficiency (CE), drug loading (DL), and in vitro drug release were carried out as previously described in [15].

### 2.5. Animals and housing

The Animal Care Unit, Medical Research Institute, Alexandria University, Egypt, provided 25 male Wistar albino rats (11–12 weeks old) weighing 160–180 g. Rats were housed in well-ventilated stainless steel wire cages, kept on a basal diet and tap water *ad libitum*, and preserved under constant laboratory conditions (temperature: 22±3°C, photoperiod: 12/12-h light/dark cycle). All experimental and animal handling procedures were performed according to the guidelines approved by Alexandria University Institutional Animal Care and Use Committee (ALEXU-IACUC), a member of the International Council for Laboratory Animal Science (ICLAS) (Approval number: AU 04 19 11 23 2 02).

### 2.6. Induction of diabetes

After two weeks of acclimatization, twenty adult male rats were fed high fat diet (HFD) (42% fat, 20% protein and 38% carbohydrate, as a percentage of total kcal) for 2 weeks. Then, at a dose of 40 mg/kg BW, they were given a single intraperitoneal injection of STZ dissolved in 0.1 M sodium citrate buffer, pH 4.5 [16]. The fasting blood glucose concentrations in both plasma and urine were monitored twice a week. Rats having blood glucose levels over 250 mg/dl with the presence of urinary glucose were used for the experiments as necessary.

### 2.7. *In vivo* experimental design

For the control non-diabetic group (Gp1), 5 rats received distilled water by oral gavage once daily for 35 days. For rats induced with DM, the rats were randomly divided into 4 groups (n = 5 rats) after induction of DM at 3 days post-STZ injection as follows: Diabetic untreated group (Gp2); Diabetic + Q-LNP at a dose of 50 mg/kg BW/day (Gp3) [1]; Diabetic + BCA at a dose of 10 mg/kg BW/day (Gp4) [13]; Diabetic + TA extract at a dosage of 200 mg/kg BW/day (Gp5) [8]. The doses were chosen based on the results of previous studies that showed these doses to be effective, as well as on the safety profiles of the compounds [1,8,13]. Rats in diabetic-treated groups (Gp3, Gp4, and Gp5) were gavage-treated orally once daily for 35 days.

### 2.8. Morris water maze (MWM)

As stated by Njan et al. [17], rats’ cognitive performance was examined four weeks following STZ injection using MWM test. A circular tank (180 cm in diameter, 60 cm in height) was filled to a depth of 30 cm with opaque water (22 ± 2°C). The pool was divided into four equal-sized quadrants (northeast, northwest, southwest, and southeast). Following that, in all experimental groups, a platform (12.5 cm in diameter) was submerged 2.0 cm below the water surface in the middle of one of the quadrants. As start points, four evenly spaced locations around the tank’s perimeter were used at the intersection of the quadrants. All rats and equipment were applied in water for training. The rats were then taught to conduct four trials every day for six days in a row. After mounting the platform, rats were allowed to remain on it for 30 seconds. The rats were treated to a probing experiment to assess their memory in 60 seconds after the platform was removed on the sixth day. The total time spent in quadrants other than the platform was examined and recorded for each rat as a measure of memory deterioration.

### 2.9. Collection of blood and tissue preparation

At the end of the experiment, blood samples were obtained from the jugular veins of fasting rats under anesthesia using a mixture of ketamine (100 mg/kg) and xylazine (5 mg/kg), allowed to coagulate at room temperature, and then centrifuged at 3000 rpm for 15 minutes. Aspiration and fractionation of the supernatant sera into three Eppendorf tubes were done. The rats were sacrificed by cervical dislocation; the cerebral cortical tissues were promptly taken and cleaned in ice-cold saline. Selected pieces of cerebral cortical tissues were kept at -80 °C for RNA extraction. For histological investigation, other sections of the cerebral cortex were rapidly preserved in 10% formalin.

### 2.10. Preparation of the cerebral cortical tissue homogenate

Collected cerebral cortical tissues were minced, washed, and homogenized by Dounce glass homogenizer in PBS buffer with pH 7.4 (10% M/V). The homogenates were spun down for 10 min at 2,000 rpm. Then the supernatant was collected and kept at -20°C for further biochemical analysis [18].

### 2.11. Biochemical parameters

Serum glucose level was estimated using a viable diagnostic kit (Catalog #GL1320; Biodiagnostics, Egypt). Sandwich ELISA kits (Catalog #INS-EASIA; Linco Research, USA) were used to measure blood insulin levels as directed by the manufacturer. Total blood cholesterol (TC) and triglyceride (TG) levels were measured using Sigma Chemical Co, USA commercial kits (TC: Catalog #MAK043; TG: Catalog #TR0100). Thiobarbituric acid reactive substances (TBARS) were used to quantify lipid peroxidation in the brain using the Ohkawa et al. [19] technique. The concentration of nitric oxide (NO) was measured calorimetrically using commercial kits (Catalog #BIO-110A, Bio Systems S.A.). Reduced glutathione (GSH) was spectrophotometrically measured at 412 nm using the technique described by Jollow et al. [20]. The activity of superoxide dismutase (SOD; EC: 1.15.1.1) and glutathione peroxidase (GPx; EC: 1.11.1.9) in brain tissues were measured using the methods of Nishikim et al. [21] and Rotruck et al. [22]. Acetylcholine was measured using the rat immunoassay kits’ manufacturer’s instructions (Ach: Catalog # MBS262132; MyBioSource, San Diego, USA). Acetylcholinesterase (AChE; EC 3.1.1.7) and Monoamine oxidase (MAO; EC 1.4.3.4) activity was evaluated in brain using ELISA kits (AChE: Catalog #MBS2880201; MAO: Catalog #MBS035703; MyBioSource, San Diego, USA). The inflammatory cytokines TNF-α and NF-kB were estimated by ELISA referring to the manufacturer’s directions for the corresponding rat immunoassay kits (TNF-α: Catalog #RAB0480; Sigma Aldrich chemical company, USA) (NF-kB: Catalog #MBS453975; MyBioSource, San Diego, USA).

### 2.12. Molecular analysis

Total RNA was isolated from brain tissue using RNA-spinTM Total RNA extraction Kit (Catalog #17211; iNtRON Biovision, Egypt Co.) following the kit’s instructions. Inducible nitric oxide synthase (iNOS), Interleukin-1β (IL-1β), Amyloid precursor protein (APP), Presenilin 2 (PSEN2), β-secretase (BACE), insulin receptor (IR), phosphoinositide 3-kinase (PI3K), Peroxisome proliferator-activated receptor gamma (PPAR-γ), Forkhead Box 1 (FOXO-1), thymoma viral oncogene (AKT) and AMP-activated protein kinase (AMPK) gene expressions were analyzed by quantitative reverse transcription-PCR (RT-PCR; Illumina, San Diego, California; Catalog #EC-900-1001)) using a real-time SYBR Green gene expression assay kit (Catalog #204056; QIAGEN, Germany). qPCR was performed in a reaction volume of 25 μL referring to the kit instructions. The specific primer sets are illustrated in Table 1. The thermal cycling conditions were as follows: initial denaturation at 95°C for 10 min, 40 cycles of amplification, each at 95°C for 60 sec, at the annealing temperature for 60 sec, and at 72°C for 60 sec. The annealing temperature and time were optimized for each primer/template combination. The expression levels were normalized to that of β-actin as an internal standard. All PCR procedures were proceeded in triplicates. All data were calculated using the 2ΔΔCt method.

**Table 1:** Primers used in the gene expression study.

Gene	Primer sequences	Accession number	Annealing temperature
iNOS	F:5’-TGGGAATGG ACTGTCCCAG-3’ R:5’-GGGATCTGAATGTGATGTTTG-3’	NM_012611	59.9°C
IL-1β	F: 5’-CCACCTCCA GGGACAGGATA-3’ R: 5’-TGGGATCTA CACTCTCCAGC-3’	NM_031512.2	60.2°C
APP	F: 5′-TGCTGAAGA TGTGGGTTCGA-3’ R:5′-GACAATCAC GTTGCTATGACAA-3′	NM_019288	59.8°C
PSEN2	F: 5′-GAGCAGAGC CA AATCAAAGG-3′ R: 5′-GGGAGAAAG AACAGCTCGTG -3`	NM_031087	59.4°C
BACE	F: 5′-CGGGAGTGG TATTATGAAGTG-3′ R: 5′-AGGATGGTGATGCGGAAG-3′	NM_019204	59.0°C
IR	F: 5′-GTCTTCGAGAACGGATCGAG-3′ R: 5′-CATGTCGGAAGAAGCAGTGA-3′	NM_017071.2	58.6°C
PI3K	F: 5′-CCAGACTCTCCCAAAAGCAG-3′ R: 5′-AGGAGTTCCACCAAGGGACT-3′	AB009636.1	58.2°C
AKT	F: 5′-ACTCATTCCAGACCCACGAC-3′ R: 5′-TGAGCTCGAACAGCTTCTCA-3′	NM_033230.3	57.8°C
PPAR-γ	F: 5′-CCCTGGCAA AGCATTTGTAT-3′ R: 5′-GAGGCCAGC ATGGTGTAGAT-3′	NM_001145367.1	57.4°C
FOXO 1	F: 5′-TATTGAGCG CTTGGACTGTG-3′ R: 5′-CACATAACC TGTGGGTGCTG-3′	NM_001191846.2	57.0°C
AMPK	F: 5′-GTCACAGGC ACATGGTTGTC-3′ R: 5′-GCATCAGCA GAGTGGCAATA-3′	NM_023991.1	56.6°C
β-Actin	F: 5′-AGGCCGGCT TCGCGGGCGA-3′ R: 5′- TGCTCCTCA GGGGCCACACG-3′	KJ696744.1	56.2°C

### 2.13. Western-blot analysis

Total protein was extracted using the ReadyPrepTM protein extraction kit provided by Bio-Rad Inc. California, USA (Catalog #163–2086) and then applied to each sample of the homogenized tissues. Protein levels were determined in each sample by Bradford Protein Assay Kit (Catalog #SK3041; CliniSciences, Nanterre—France) for quantitative protein analysis which was provided from Bio Basic Inc. California, USA (Markham Ontario L3R 8T4 Canada). 20 μg protein concentrations from each sample was then loaded with an equal volume of 2x Laemmli sample buffer containing 4% SDS, 10% 2-mercaptoehtanol, 20% glycerol, 0.004% bromophenol blue and 0.125 M Tris HCl. The pH was checked and brought to 6.8. Before loading on polyacrylamide gel electrophoresis, each prior combination was heated at 95°C for 5 minutes to achieve protein denaturation.

Samples were separated on a polyacrylamide gel; the procedure was abbreviated as SDS-PAGE form Sodium Dodecyl Sulfate PolyAcrylamide Gel Electrophoresis, which is a standard technique for separating proteins according to their molecular weight. Polyacrylamide gels were created with the Bio-Rad Laboratories inc Cat # 161–0181 TGX Stain-FreeTM FastCastTM Acrylamide Kit (SDS-PAGE). The SDS-PAGE TGX Stain-Free FastCast was made according to the manufacturer’s instructions. From bottom to above, the gel was built in a transfer sandwich (filter paper, PVDF membrane, gel and filter paper). The sandwich was placed in the transfer tank with 1x transfer buffer, which is composed of 25 mM Tris and 190 mM glycine and 20% methanol. The blot was then run for 7 minutes at 25 V to allow protein bands to transfer from gel to membrane using the BioRad Trans-Blot Turbo.

The membrane was blocked in Tween 20 (TBST) buffer with tris-buffered saline and 3% bovine serum albumin (BSA) at room temperature for 1 hr. The components of blocking buffer were as follow; 20 mM Tris pH 7.5, 150 mM NaCl, 0.1% Tween 20 and 3% bovine serum albumin (BSA). Primary antibody of GSK-3β (Catalog #sc-81462, Santa Cruz Biotechnology, Inc. Europe) was purchased. Primary antibody was diluted in TBST. At 4°C, each 1ry antibody solution was incubated overnight against the blotted target protein. The blot was washed with TBST 3–5 times for 5 minutes. For 1 hour at room temperature, the blotted target protein was incubated in the HRP-conjugated 2ry antibody solution (Goat anti-rabbit IgG- HRP-1mg Goat mab -Novus Biologicals). The blot was washed with TBST 3–5 times for 5 minutes.

The chemiluminescent substrate (ClarityTM Western ECL substrate Bio-Rad cat#170–5060) was used on the blot as directed by the manufacturer. In brief, equal amounts of solution A (Clarity Western luminal/enhancer solution) and solution B were combined (peroxidase solution). A CCD camera-based imager was used to collect the chemiluminescent signals. On the ChemiDoc MP imager, image analysis software was used to read the band intensity of the target proteins against the control sample beta actin (housekeeping protein) by protein normalization.

### 2.14. Histological preparation

After fixation of brain samples in formalin solution (10%), samples were treated with conventional grades of alcohol and xylol, embedded in paraffin and sectioned at 4 to 6 μm thickness. The sections were stained with Hematoxylin and Eosin (H&E) [23].

### 2.15. Immunohistochemistry assay “GFAP”

Immunohistochemistry was carried out using the approach previously reported by Diene et al. [24]. Following a 10-minute incubation with 3% H_2_O_2_, sections were blocked for 15 minutes at room temperature with 2% bovine serum albumin (BSA) in PBS containing 0.4% Triton X-100 (PBSTx, Sigma Chemical Co., USA), and then incubated for 48 hours at 4°C with polyclonal glial fibrillary acidic protein (GFAP) antiserum raised in rabbit (Z0334 Dako, 1:500, UK). After numerous washes with PBS-Tx, the sections were incubated at room temperature for 2 hours with biotin-conjugated goat anti-rabbit IgG secondary antibody (Amersham, 1:50, UK). After washing again, sections were incubated with horse radish peroxidase (HRP)-conjugated streptavidin at 37°C for 15 min. At the end of incubation, sections were stained by 3,3-diaminobenzidine (DAB, K3467, Dako) at room temperature for 15 min in the dark. All sections were examined under a light microscope (Olympus IX70, Olympus Optical Co. Ltd, Japan). Image J software was used to perform semi-quantitative densitometric analysis on the intensity of GFAP immunoreactivity.

### 2.16. Statistical analysis

Statistical analysis was performed using the SPSS software package (Version 16.0). Data are provided as mean ± SE. One-way analysis of variance (ANOVA) was used to compare groups, followed by Least Significant Difference (LSD) at p<0.05 significance [25].

## 3. Results

### 3.1. HPLC results of isolated Q and BCA

**Fig 1:**
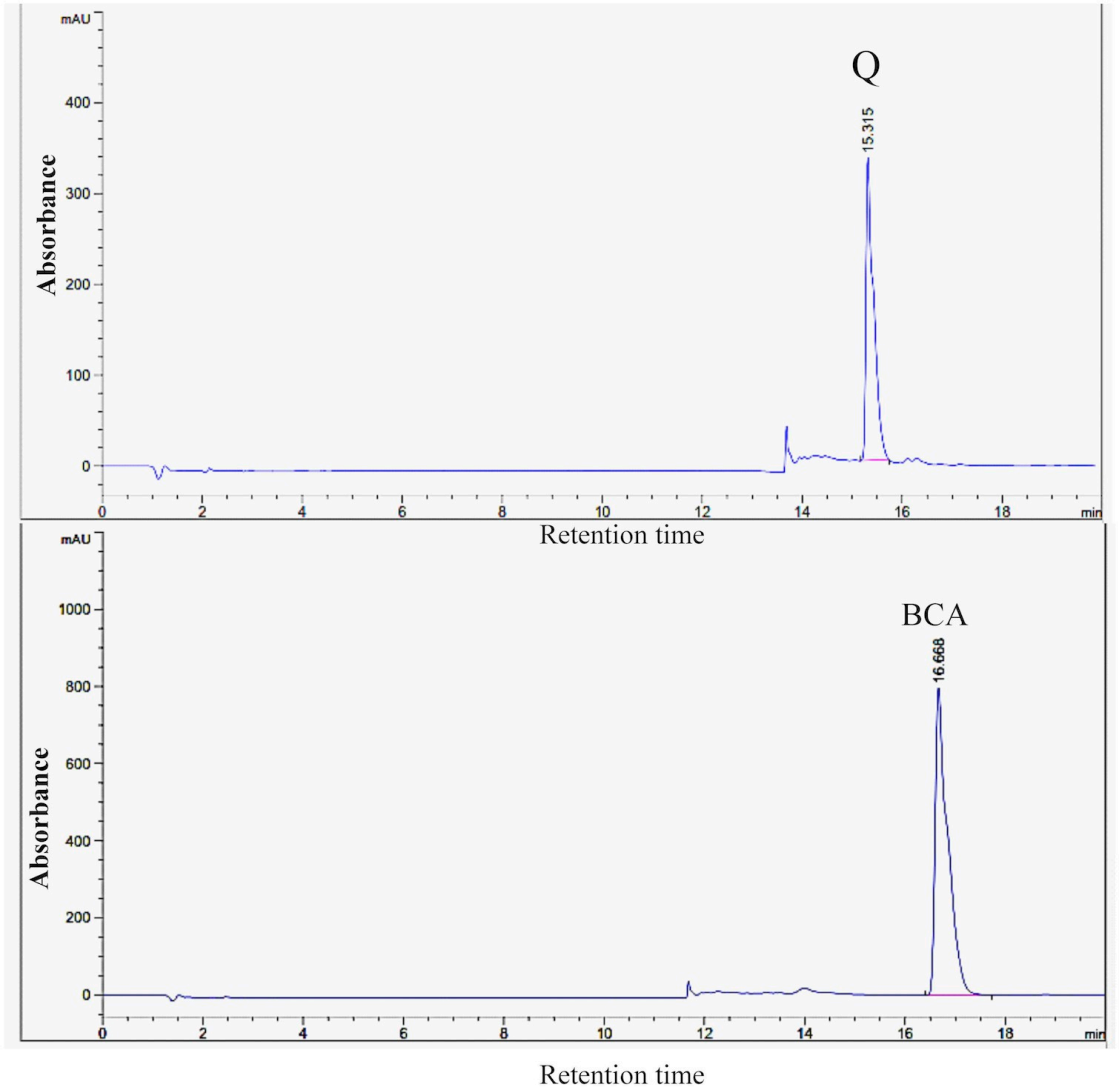
HPLC results of isolated Q and BCA.

Q and BCA were the main constituents recovered from the TA extract. Q was identified and quantified using HPLC at 0.2% with purity above 97%, followed by BCA at 0.1% with purity exceeding 98% (Fig 1).

### 3.2. Physicochemical characterization of Q-LNP

**Fig 2:**
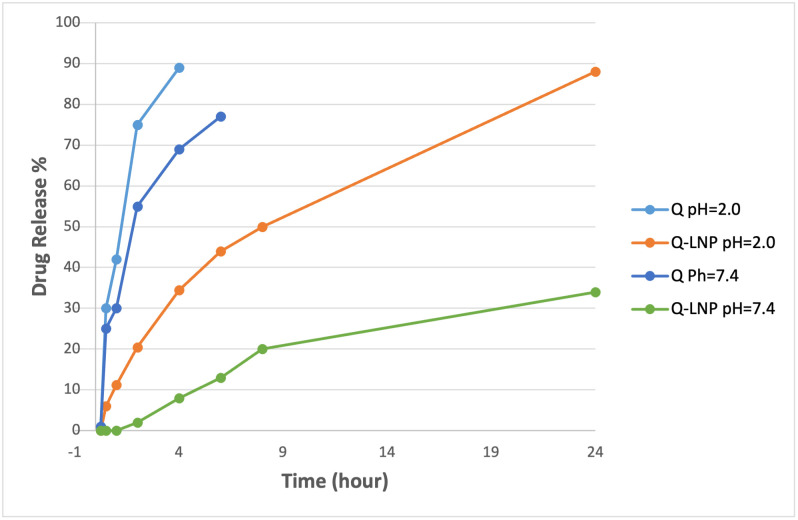
*In vitro* release of Q and Q-LNP at both pH 7.4 and 2.0 using 100 mL of phosphate-buffered solution and diluted HCl respectively as a release medium at 37ºC, 100 rpm.

As shown in Table 2, the particle size and PDI of Q-LNP were 200 nm and 0.20 with a zeta potential +14.3mV. The drug loading of the conjugates was estimated by determining the amount of unbound drug using an HPLC method as described previously in [1]. Each measurement was repeated three times. The *in vitro* release profile of Q and Q-LNP was investigated at pH 7.4 (PBS) and pH 2 (diluted HCl solution) using a dialysis bag method (Fig 2). Q was almost completely released after 6 hr. at pH 2.0 and 7.4. On the other hand, Q release from Q-LNP showed a slow sustained release of 34% over 24 hr at pH 7.4, with an accelerated release at pH 2.0 (88%) due to easier hydrolysis of the ester bond in acidic medium.

**Table 2:** Physicochemical characterization of Q-LNP.

	PS (nm)	PDI	ζ (mV)	CE (mg/wt%)	DL (wt%)
**Q-LNP**	200± 9.72	0.20 ± 0.002	+14.3±0.75	6.7/41	6.1

Particle size (PS), polydispersity index (PDI), zeta potential (ζ), conjugation efficiency (CE), drug loading (DL) (n = 3).

### 3.3. Cognitive performance alterations in different experimental groups

**Fig 3:**
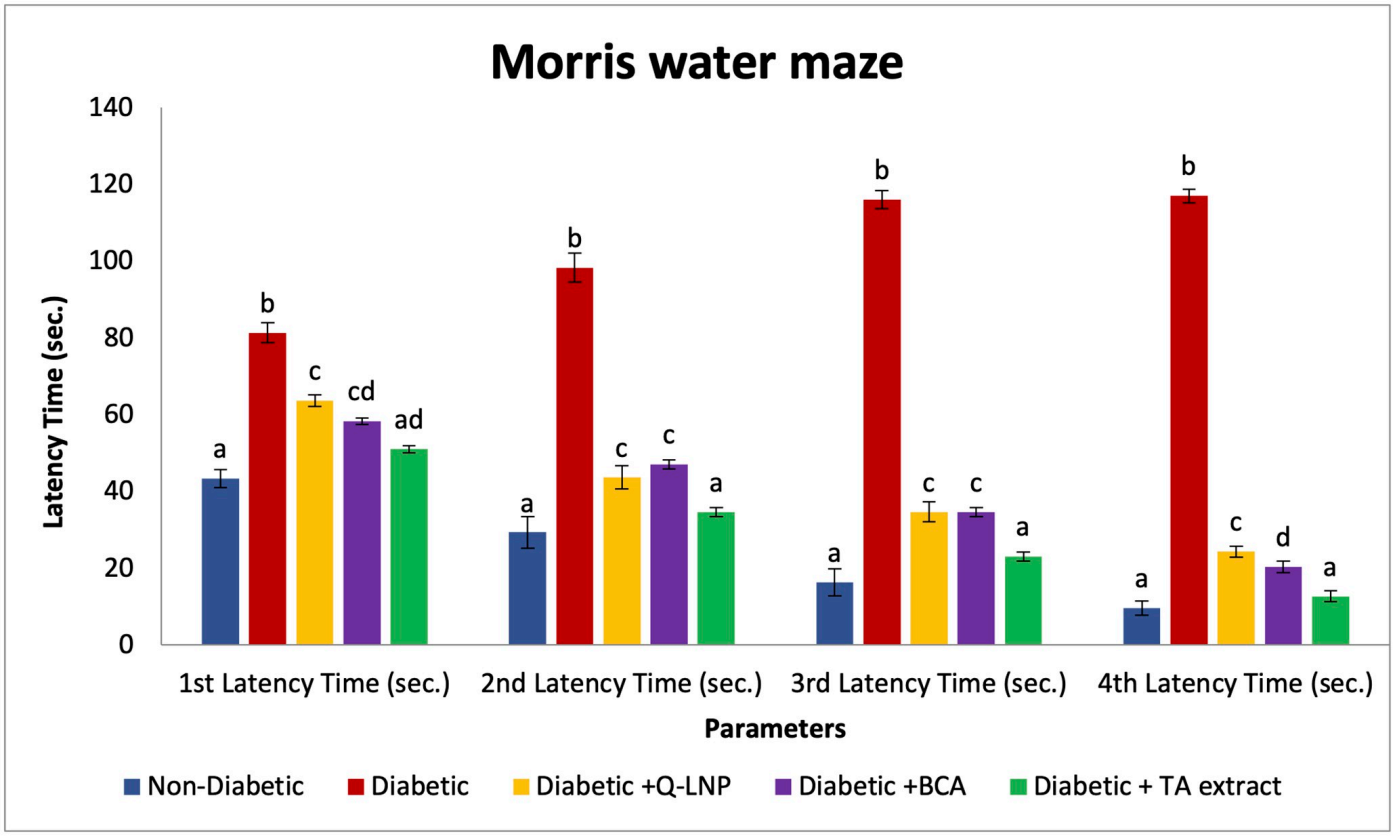
Changes in the cognitive performance in different experimental groups. Values are expressed as means ± SE (n = 5). Mean values having different superscript letters a, b, c, and d are significantly different from each other (p < 0.05).

Changes in the cognitive performance in different experimental groups are illustrated in Fig 3. Diabetic untreated rats showed a significant delay in the latency time in locating the platform over the control group. In contrast, diabetic rats supplemented with Q-LNP, BCA, and TA extract revealed significant reduction in the latency time to trace the platform by improving the choice of spatial swimming strategy rather than systemic and looping strategies. Moreover, all the treated groups showed substantial increases in the latency time compared to the control group except for the Diabetic+TA extract group.

### 3.4. Evaluation of the serum levels of glucose, insulin, TC and TG

The data in Table 3 reveal that the serum of diabetic untreated rats had a significant rise in glucose, TC, and TG levels while causing a significant drop in insulin levels as compared with the normal group. On the other hand, diabetic rats administered Q-LNP, BCA, and TA extract showed a significant improvement in these parameters as compared with the diabetic untreated group. Overall, the maximum enhancement of these parameters was observed in the Diabetic+TA extract group.

**Table 3:** Changes in the serum levels of glucose, insulin, total cholesterol (TC) and triglyceride (TG) in different experimental groups.

Parameters	Experimental groups				
	Non-Diabetic	Diabetic	Diabetic + Q-LNP	Diabetic + BCA	Diabetic + TA extract
Glucose (mg/dl)	96.0±3.78^a^	339.3±14.4^b^	151.0±0.57^c^	145.0±3.05^c^	125.3±2.60^d^
Insulin (mU/L)	1.12±0.04^a^	0.59±0.02^b^	0.84±0.01^c^	0.92±0.01^d^	0.97±0.01^e^
TC (mg/dl)	63.6±2.72^a^	103.3±5.54^b^	76.7±5.17^c^	69.6±2.6^ac^	57.66±5.36^a^
TG (mg/dl)	62.3±1.85^a^	101.3±4.97^b^	71.6±1.76^c^	78.3±2.60^c^	63.3±2.40^a^

Values are expressed as means ± SE (n = 5).

Mean values, in the same row, having different superscript letters a, b, c and d are significantly different from each other (p < 0.05).

### 3.5. Evaluation of the cerebral cortical levels of oxidant and antioxidant profiles

Table 4 shows the changes in the oxidant and antioxidant profiles in the cerebral cortex of different experimental groups. Diabetic untreated rats demonstrated significantly increased levels of brain TBARS and NO, whereas the levels of GSH and the activities of SOD and GPx were dramatically decreased. In contrast, diabetic rats that were treated with Q-LNP, BCA, and TA extract exhibited a significant diminution in the levels of TBARS and NO but a significant rise in the levels of GSH and associated enzymes activities of SOD and GPx. The maximum enhancement of these parameters was observed in Diabetic+TA extract group.

**Table 4:** Changes in the oxidant and antioxidant profile in the cerebral cortex of different experimental groups.

Parameters	Experimental groups				
	Non-Diabetic	Diabetic	Diabetic + Q-LNP	Diabetic + BCA	Diabetic + TA extract
TBARS (nM/ mg protein)	11.60±0.37^a^	31.53±1.03^b^	21.41±0.74^c^	17.66±0.44^d^	15.06±0.52^e^
NO (μM/ mg protein)	18.53±0.58^a^	47.73±0.67^b^	21.56±0.43^c^	25.13±0.82^d^	19.53±0.52^a^
GSH (mg/ mg protein)	6.01±0.14^a^	2.55±0.18^b^	4.26±0.23^c^	5.20±0.21^d^	5.83±0.22^a^
SOD (U/mg protein)	4.76±0.07^a^	1.93±0.03^b^	4.69±0.10^a^	3.80±0.05^c^	4.72±0.16^a^
GPX (U/mg protein)	30.56±1.06^a^	12.06±0.49^b^	26.56±0.40^c^	26.60±0.62^c^	30.63±0.52^a^

Values are expressed as means ± SE (n = 5).

Mean values, in the same row, having different superscript letters a, b, c, d and e are significantly different from each other (p < 0.05).

### 3.6. Changes in the neurochemical parameters in the cerebral cortex

Several neurotransmitters and related enzymes were investigated, including acetylcholine (Ach), dopamine (DA), serotonin (5-HT), acetylcholinesterase (AChE), and monoamine oxidase (MAO). Results depicted in Table 5 summarize the changes in the neurotransmitters in the cerebral cortex of different experimental groups. Diabetic untreated rats showed a substantial decrease in the levels of Ach, DA, and 5-HT, whereas the levels of AChE and MAO were significantly increased as compared with the control group. However, diabetic rats that were supplemented with Q-LNP, BCA, and TA extract revealed considerably enhancement in the levels of cortical Ach, DA, and 5-HT and a significant decrease in the activities of cortical AchE and MAO when compared to the diabetic group. Interestingly, treatment with TA extract normalized the DA, 5-HT, AchE and MAO levels back to similar levels as the control. Furthermore, Diabetic+Q-LNP achieved the normalization of DA, 5-HT and MAO to control levels as well.

**Table 5:** Changes in the neurochemical parameters in the cerebral cortex of different experimental groups.

Parameters	Experimental groups				
	Non-Diabetic	Diabetic	Diabetic + Q-LNP	Diabetic + BCA	Diabetic + TA extract
Ach (pmol/mg protein)	9.46±0.17^a^	4.40±0.15^b^	8.53±0.20^c^	6.67±0.08^d^	8.30±0.11^c^
DA (μg/ mg protein)	270.0±7.23^a^	106.3±4.41^b^	265.6±7.83^a^	232.6±6.74^c^	264.03±8.08^a^
5-HT (μg/ mg protein)	116.6±3.75^a^	63.6±2.90^b^	113.6±4.48^a^	96.6±3.52^c^	116.3±2.72^a^
AchE (U/ mg protein)	12.06±0.12^a^	17.87±0.20^b^	13.40±0.17^c^	14.5±0.15^d^	12.40±0.25^a^
MAO (Mu/ mg protein)	25.07±0.58^a^	44.07±1.36^b^	27.27±0.67^a^	31.90±1.15^c^	27.20±0.32^a^

Values are expressed as means ± SE (n = 5).

Mean values, in the same row, having different superscript letters a, b, c and d are significantly different from each other (p < 0.05).

### 3.7. Role of Q-LNP, BCA, and TA extract on the cerebral cortical inflammatory cytokines

Diabetic untreated rats revealed a significant elevation in the levels of TNF-α and NF-κB as compared to the non-diabetic group. Alternatively, co-administration of Q-LNP, BCA, and TA extract caused significantly diminished levels of TNF-α and NF-κB (Table 6). It was observed that Diabetic+Q-LNP achieved the normalization of NF-κB level to reach control levels.

**Table 6:** Changes in the inflammatory markers in the cerebral cortex of different experimental groups.

Parameters	Experimental groups				
	Non- Diabetic	Diabetic	Diabetic + Q-LNP	Diabetic + BCA	Diabetic + TA extract
TNF-α (Pg/mg protein)	45.30 ±0.51^a^	92.00±0.34^b^	49.10±0.23^c^	65.26±0.21^d^	47.1±0.10^e^
NF-κB (Pg/mg protein)	43.00±0.34^a^	97.76±0.43^b^	42.63±0.32^a^	50.00±0.17^c^	45.13±0.35^d^

Values are expressed as means ± SE (n = 5).

Mean values, in the same row, having different superscript letters a, b, c, d and e are significantly different from each other (p < 0.05).

### 3.8. Role of Q-LNP, BCA, and TA extract on gene expression levels of iNOS, IL-1β, APP, PSEN2, BACE, IR, PI3K, PPAR-γ, FOXO-1, AKT, and AMPK

Table 7 shows the significant increases in the levels of iNOS, IL-1 β, APP, BACE, and PPAR-expression in the cerebral cortex of diabetic rats compared with the control group. Conversely, when diabetic rats were treated with Q-LNP, BCA, and TA extract, their expression of these genes were improved. Furthermore, the expression of PSEN2, IR, PI3K, FOXO-1, AKT, and AMPK were substantially lower in diabetic untreated rats as compared to the control group, while the expression of these genes increased significantly post exposure to Q-LNP, BCA, and TA extract as compared to the diabetic untreated rats. Overall, the maximum enhancement of these parameters was observed in the Diabetic+TA extract group.

**Table 7:** Changes in the gene expression levels of inducible nitric oxide synthase (iNOS), Interleukin-1β (IL-1β), Amyloid precursor protein (APP), Presenilin 2 (PSEN2), β-secretase (BACE), insulin receptor (IR), phosphoinositide 3-kinase (PI3K), Peroxisome proliferator-activated receptor gamma (PPAR-γ), Forkhead Box 1 (FOXO-1), thymoma viral oncogene (AKT) and AMP-activated protein kinase (AMPK) in the cerebral cortex of different experimental groups.

Parameters	Experimental groups				
	Non- Diabetic	Diabetic	Diabetic + Q-LNP	Diabetic + BCA	Diabetic + TA extract
iNOS	1.00±0.04^a^	3.38±0.12^b^	1.90±0.03^c^	1.51±0.01^d^	1.37±0.02^d^
IL-1β	1.00±0.03^a^	5.43±0.11^b^	2.60±0.19^c^	1.97±0.02^d^	1.46±0.03^e^
APP	1.00±0.03^a^	5.11±0.09^b^	2.31±0.12^c^	2.02±0.02^d^	1.53±0.02^e^
PSEN2	1.00±0.03^a^	0.54±0.01^b^	0.75±0.1^c^	0.84±0.02^d^	0.96±0.01^a^
BACE	1.00±0.02^a^	4.05±0.16^b^	1.64±0.20^c^	1.81±0.03^d^	1.63±0.01^c^
IR	1.00±0.03^a^	0.74±0.02^b^	0.97±0.06^c^	0.84±0.01^d^	1.30±0.01^e^
PI3K	1.00±0.01^a^	0.24±0.002^b^	0.93±0.05^c^	0.76±0.01^d^	0.99±0.01^c^
PPAR-γ	1.00±0.06^a^	5.22±0.04^b^	2.50±0.08^c^	1.91±0.12^d^	1.19±0.01^e^
FOXO-1	1.00±0.01^a^	0.43±0.01^b^	0.76±0.01^c^	0.60±0.03^d^	0.93±0.01^e^
AKT	1.00±0.03^a^	0.12±0.003^b^	0.75±0.02^c^	0.80±0.01^cd^	0.85±0.02^d^
AMPK	1.00±0.07^a^	0.04±0.0002^b^	0.79±0.02^c^	0.65±0.01^d^	0.80±0.01^e^

Values are expressed as means ± SE (n = 5).

Mean values, in the same row, having different superscript letters a, b, c, d and e are significantly different from each other (p < 0.05).

### 3.9. Role of Q-LNP, BCA, and TA extract on the cerebral cortex GSK-3β

**Fig 4:**
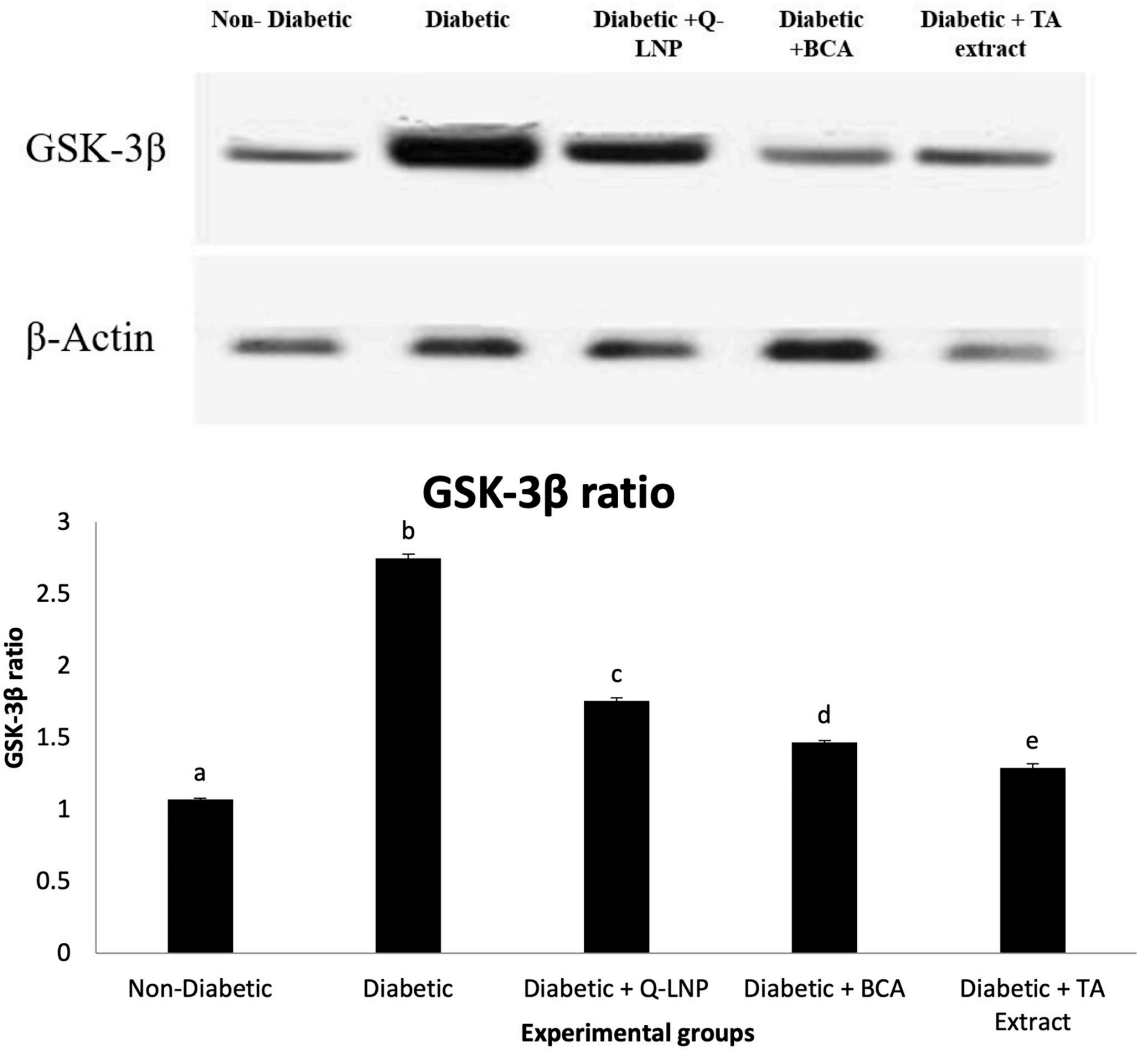
Changes in the glycogen synthase kinase-3β (GSK-3β) in the cerebral cortex of different experimental groups. Values are expressed as means ± SE (n = 5). Mean values having different superscript letters a, b, c, and d are significantly different from each other (p < 0.05).

When compared to the control group, diabetic induction resulted in a significant elevation in GSK-3β levels in the cerebral cortex, while supplementation of Q-LNP to diabetic rats significantly lowered levels of GSK-3β in the brain tissue compared to the diabetic untreated group. In addition, the levels of GSK-3β in the brain tissues of BCA and TA treated rats were also considerably lower than that in the diabetic group (Fig 4). Generally, the maximum enhancement of the expression of Gsk-3β was observed in the Diabetic+TA extract group, followed by the Diabetic+BCA group.

### 3.10. Histopathological examination of the cerebral cortex

**Fig 5:**
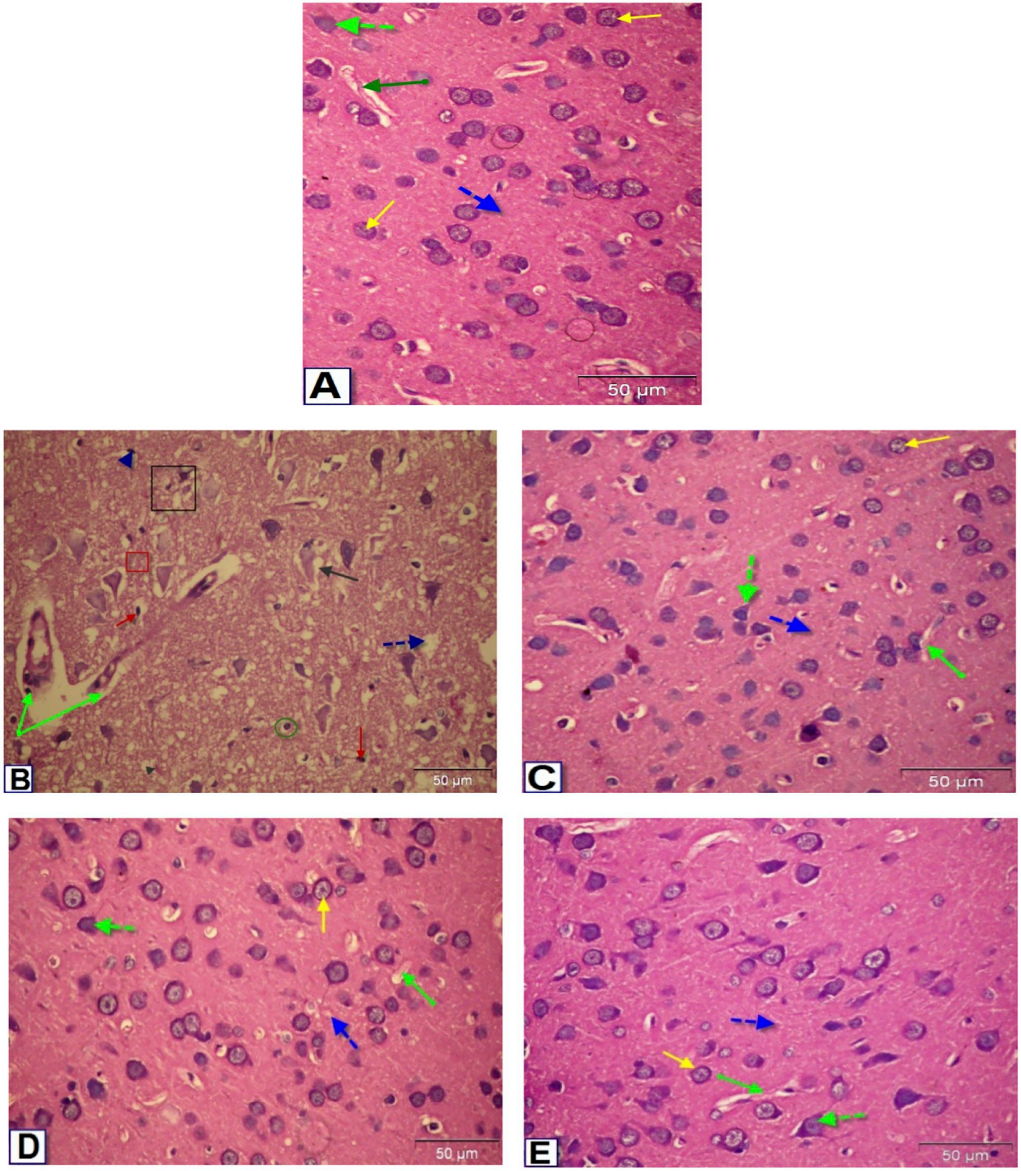
Photomicrographs of sections in the cerebral cortex of male rats. (A) Control rats showing normal structure of the cerebral cortex; normal pyramidal cell (green dotted arrow) and granule cell with pale open face nucleus (yellow arrow) normal blood vessel (green arrow). (B) Diabetic rats showing pericellular vacuoles (black arrow), dilatation of blood capillary with congestion (green arrow), neuronal degeneration with pyknotic nuclei (black square), encephalomelacia (red square), dark degenerated neuron (red arrow), pyknotic nuclei (blue head arrow), vacuolated neuropil (blue dotted arrow) and glial cell (green circle). (C, D, E): sections of the cerebral cortex of Diabetic+Q-LNP, Diabetic+BCA, and Diabetic+TA extract treated groups, respectively, showing slight improvement of the neuronal cells (H&E, 400X).

Microscopic examination of the nondiabetic control group brain sections through the cerebral cortex (Fig 5A) revealed normal histo-architecture of the cerebral cortex tissue, with pyramidal cells of cerebral cortex having triangular shape with dendritic spines and granule cells, and densely packed small neurons revealing normal appearance. Alternatively, diabetic untreated rats showed pericellular vacuoles, blood capillary dilatation with congestion, neuronal degeneration with pyknotic nuclei, encephalomelacia, dark degenerated neuron, vacuolated neuropil, and glial cells (Fig 5B). Fig 5C–5E displayed sections of the cerebral cortex from the Diabetic+Q-LNP, Diabetic+BCA, and Diabetic+TA extract treated groups, respectively, showing a slight improvement in neuronal cells.

### 3.11. Immunohistochemistry of GFAP

**Fig 6:**
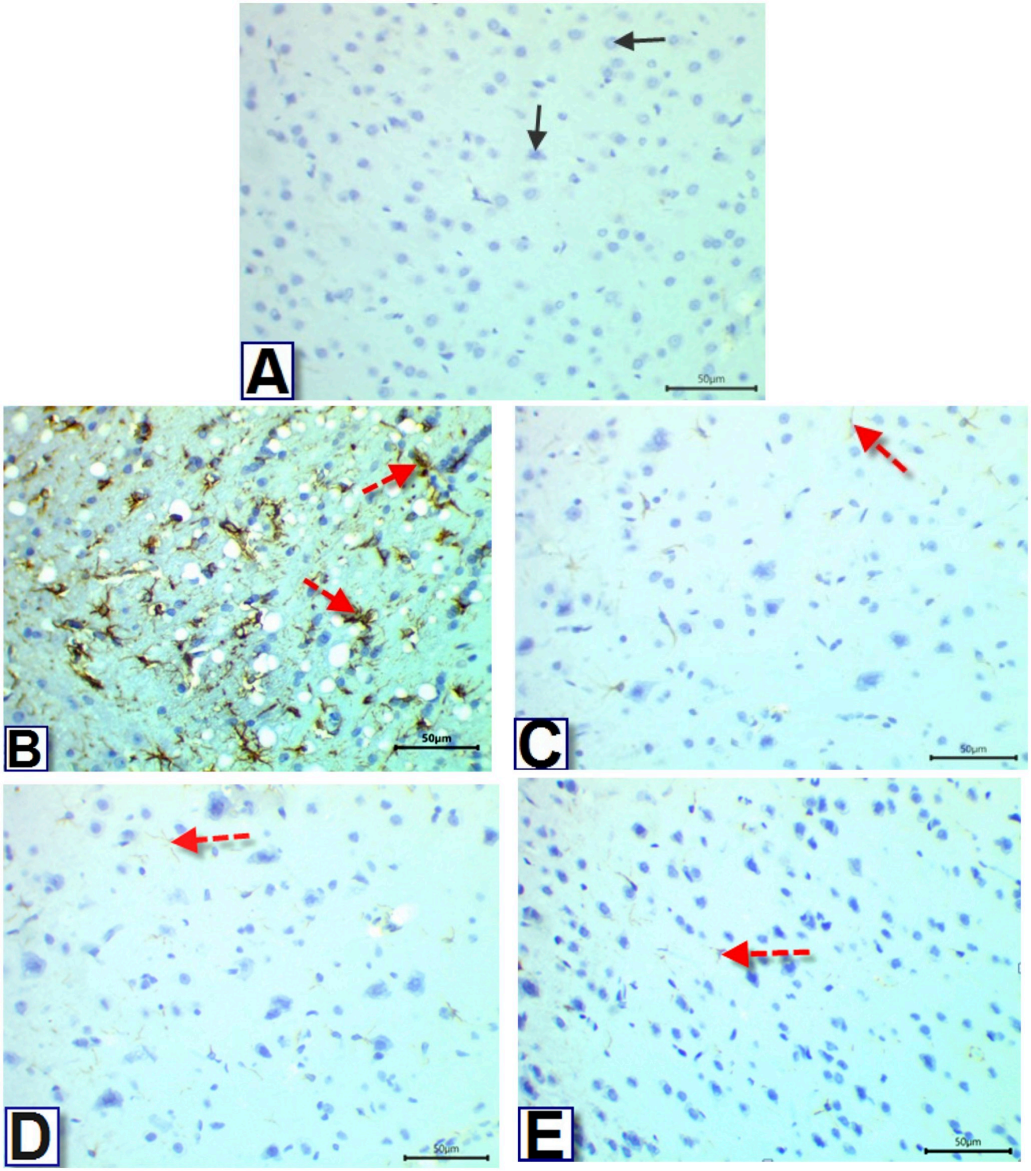
Photomicrographs of sections in rat’s cerebral cortex, showing immunoreactivity for GFAP. Sections in control group (A) showed negative reaction for GFAP, while cerebral cortex sections from the untreated-diabetic group (B) showed strong reaction for GFAP. Sections from Diabetic+Q-LNP, Diabetic+BCA, and Diabetic+TA extract treated groups (C, D, & E), respectively, showed reduced number and intensity of GFAP positive cells (GFAP, 400X).

**Fig 7:**
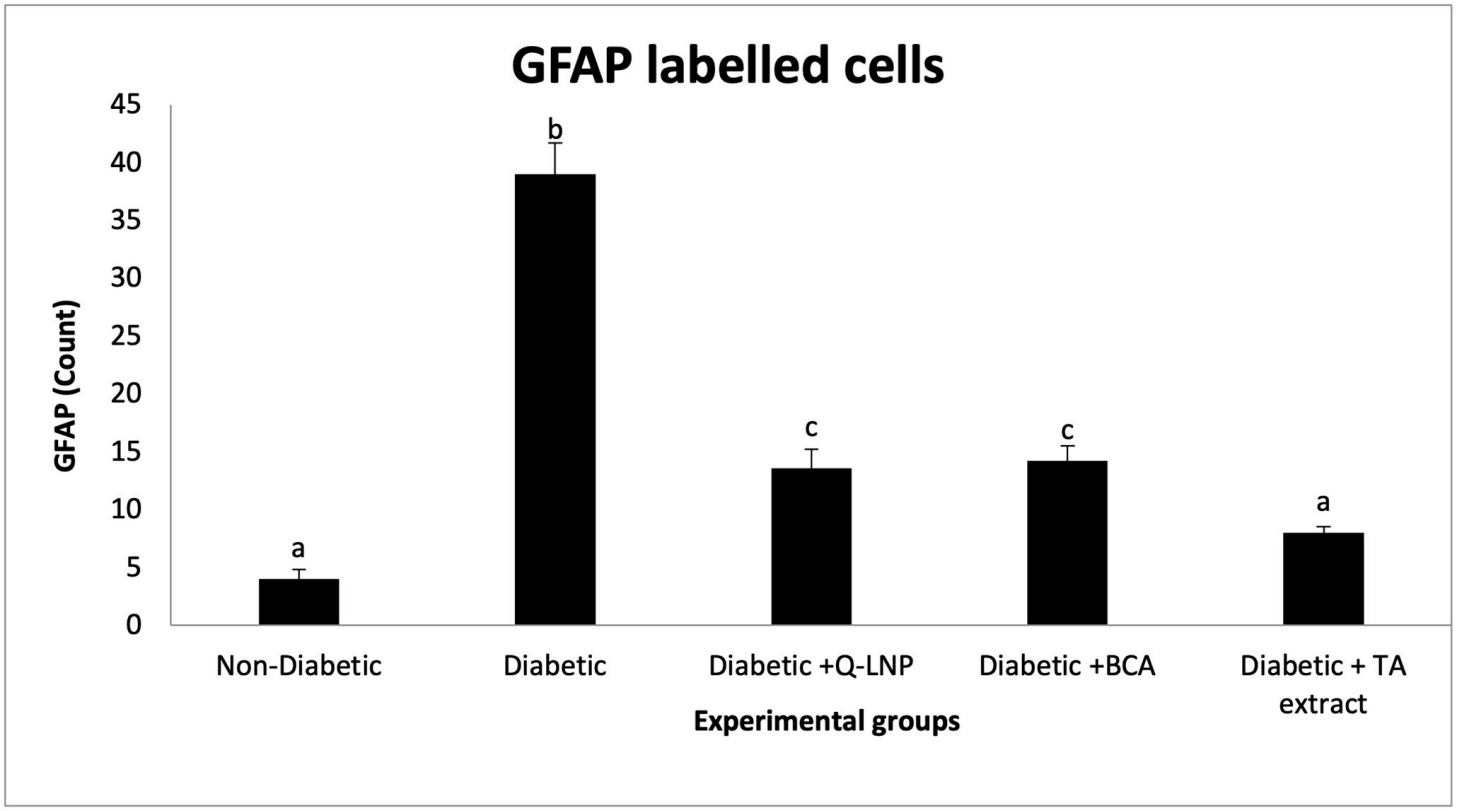
Changes in the mean number of GFAP labeled cells in the cerebral cortex of male rats in the different experimental groups. Values are expressed as means ± SE (n = 5). Mean values having different superscript letters a, b, c, and d are significantly different from each other (p < 0.05).

Immunohistochemical studies revealed a rise in the number of astrocytes with positive GFAP immunostaining in the cerebral cortex of diabetic rats compared to nondiabetic controls (Figs 6 and 7), while administering Q-LNP, BCA, and TA extract to diabetic rats significantly reduced the number of GFAP-positive astrocytes relative to the non-treated diabetic group. Notably, administration of TA extract to diabetic rats normalized the number of astrocytes stained with GFAP to return to control levels.

## 4. Discussion

A growing body of evidence suggests a link between diabetes and neurodegenerative diseases. This link is thought to be due to a number of factors, including hyperglycemia, OS, and inflammation [4]. The current diabetic model was linked to significant alteration on cognitive functions measured in the MWM test. Diabetic rats showed a significant delay in the latency time to locate the platform compared to non-diabetic rats. Previous studies have shown that diabetic rats have impaired performance on the MWM test [26,27]. They typically have longer latencies to find the platform, make more errors, and take longer paths to the platform. The exact mechanisms by which diabetes impairs MWM performance are not fully understood. However, it is thought that hyperglycemia may play a role [27]. Increasing evidence points to neuroinflammation as a key factor in the emergence of behavioral and cognitive impairments caused by diabetes [26]. We investigated the effect of treating diabetic rats with Q-LNP, BCA, and TA extract with respect to cognitive performance. Results showed that these treatments caused improvement in the cognitive impairment by shortening the latency time to locate the platform. These results are consistent with those of Bhutada et al. [7], who found that Q ameliorated MWM test performance in STZ-induced diabetic rats. This could be due to Q’s anti-inflammatory properties which inhibit pro-inflammatory cytokines and AChE activity [27]. Furthermore, BCA has also been shown to enhance learning and memory in rats, and it has a variety of other pharmacological properties, including antioxidant, anti-tumor, and anti-hyperglycemic activity [28]. In addition, TA extract is rich in bioflavonoids, which enhance cognitive functions by targeting many enzymes and molecules in cells as a result of their anti-inflammatory, antioxidant, or signaling actions [1,29]. It was noticeable that the TA extract-treated group was more effective than other treated groups in improving spatial learning and memory. This is likely due to the synergistic effects of the different active constituents in TA extract [1,8].

The body regulates glucose production by controlling glucose and insulin levels in the blood along with hepatic insulin action. In T2DM, glucose production becomes elevated due to impaired insulin secretion and utilization. This leads to decreased glucose uptake by tissues, which further increases glucose production [13]. Our findings revealed that diabetic rats had impaired serum glucose and insulin levels. These findings are consistent with previous research that has shown that hyperglycemia is associated with increased oxidative stress, impaired insulin action, and partial destruction of pancreatic β-cells [1]. Treating diabetic rats with Q-LNP, BCA, and TA extract caused significant improvement in serum glucose and insulin levels. Consistent with the previous report, oral administration of Q-LNP improves glucose and lipid metabolism, as well as changes β-cell functions and insulin activity [1]. Moreover, BCA treatment actually controls hyperglycemia in T2DM rats with regard to the reduction in insulin resistance and increased insulin sensitivity at the cellular level [13]. Mohamed et al. [8] suggested that TA extract exerts antidiabetic effects by decreasing the elevated glucose level in diabetic rats, an effect caused by modulating the enzymes involved in glucose metabolism. Here it was noticeable that the TA extract-treated group is more effective than other treated groups in attenuating hyperglycemic parameters. These results may arise from its effectiveness in acting as an antidiabetic agent that works through a variety of mechanisms [1,8,30].

Results of the current study showed diabetic rats with considerably increased TC and TG levels due to insulin resistance at the cellular level. This result could be explained by the increase in the lipolysis cycle and decreased insulin levels [1,16]. Moreover, insulin resistance is a major contributor to the development of dyslipidemia in T2DM. This is due to an increase in free fatty acid synthesis, which in turn leads to an increase in TC and TG production [15]. However, treating diabetic rats with Q-LNP, BCA, and TA extract alleviated the disruption in the serum lipid profile. The present results are in accordance with Abdou et al. [30], who reported that Q reduced serum levels of TC and TG in diabetic rats. Furthermore, reducing insulin resistance is the key mechanism by which BCA treatment lowers hyperlipidemia in diabetic rats [13]. Additionally, TA extract is known for its ability to inhibit lipase activity and thus reduce fat absorption. The inhibitory action of lipase decreases the hydrolysis of dietary TGs into monoglycerides and free fatty acids, as it lowers the TC and TG in diabetic rats [8]. These findings suggest that TA extract may be a potential treatment for hyperlipidemia.

The current diabetes model is linked to increased OS, which is consistent with earlier research [1,8]. In neurodegenerative diseases, the brain is especially vulnerable to oxidative damage due to the imbalance between the generation of oxygen free radicals and antioxidant defense systems [31]. Results of our current study showed the cerebral cortex of diabetic untreated rats having considerably increased levels of TBARS and NO, whereas the levels of GSH and the activities of SOD and GPx were dramatically decreased. These results are in accordance with those of others [2,32]. One of the mechanisms that may contribute to diabetic neuronal injury is the excessive production of ROS from the auto-oxidation of elevated intracellular glucose levels [32]. Conversely, the administration of Q-LNP, BCA, and TA extract counteracts these detrimental impacts. This is because these compounds have antioxidant properties and can boost the endogenous antioxidant enzyme system [9]. Moreover, Q acts as an antioxidant that can exert neuroprotective effects [10], and BCA can reduce TBARS and increase GSH levels, hence mitigating oxidative damage [11]. Additionally, it has been reported that TA extract has antioxidant effects that can improve oxidative parameters and increase antioxidant enzymes, suggesting the protective and preventive effect against STZ action mediated through the neutralization of oxygen free radicals [8]. Generally, it was noticeable that the TA extract-treated group is more effective than other treated groups in attenuating oxidative stress parameters. In addition to the previously discussed reasons including synergistic effects and the multiple targets, it is also possible that the superior efficacy of TA in attenuating oxidative stress is due to the presence of yet unidentified compounds in the plant extract [1,8]. It is well-known that cognitive function is regulated by various neurotransmitters including ACh, DA and 5-HT. ACh is oxidized by AChE whereas DA and 5-HT are metabolized by MAO [33]. The current study showed that diabetes is associated with defects in the release of ACh, DA and 5-HT in the cerebral cortex. In agreement with our results, Rivera et al. [33] reported that a decline in ACh levels results from impairments in insulin and insulin-like growth factor I (IGF-I) signaling mechanisms in the brain. The results of the present work regarding DA and 5-HT decreases were in agreement with the study of Ezzeldin et al. [34]. The decline in DA resulted from reduction in its synthesis and turnover in the cerebral cortex, while the decrease in 5-HT levels during diabetes may be due to a chronic anabolic deficit caused by a decrease in amino acids in the brain, with the consequent decrease in 5-HT synthesis [34]. In contrast, it was of great interest to note that Q-LNP, BCA, and TA extract treatments can restore the levels of these neurotransmitters to control values. Other studies have also elaborated on the possible restoration of neurotransmitters, including Q’s ability to cause a considerable rise in 5-HT [35] and to enhance ACh by inhibiting AChE and hence boosting cognitive function improvement [10]. Likewise, BCA has been shown to protect dopaminergic neurons by inhibiting the inflammatory response and the MAPK signaling pathway [12]. Other studies showed that TA extract rescued dopaminergic neurons from neurotoxicity by activating estrogen receptor (ER)-mediated signaling pathways [36].

The results of the present study demonstrate that AChE and MAO activities were increased in the cerebral cortex of diabetic rats. These results are similar to those found by Sriraksa et al. [37]. The increase in AChE activity caused by diabetes leads to a reduction in the efficiency of cholinergic neurotransmission due to a decrease in ACh levels in the synaptic cleft, therefore leading to neurological dysfunctions associated with diabetes [37]. The alterations in AChE activity in diabetes are induced by hyperglycemia and lipid peroxidation that triggers brain dysfunction [38]. MAO activity is elevated in response to oxidative stress and hyperglycemia [37]. Our findings suggest that the increased activities of AChE and MAO in the cerebral cortex of diabetic rats may contribute to the cognitive impairment that is associated with this condition.

The treatment of diabetic rats with Q-LNP, BCA, and TA extract were able to prevent the increase in AChE and MAO activities. These results regarding AChE are similar to Maciel et al. [38] who reported that antioxidants such as Q prevent the rise in AChE activity and therefore the cognitive deficits induced by the diabetic state. A decrease in AChE activity indicates an increase of ACh levels in the synaptic cleft, enabling an improvement in cognitive functions such as learning and memory. Moreover, it has been reported that BCA prevented the rise in AChE activity induced by scopolamine [11]. TA extract is rich in flavonoids, compounds that possess notable inhibitory activity against AChE enzyme [39]. It has been demonstrated that Q exerts an inhibitory effect on AChE and MAO [37]. Available reports suggest that BCA inhibits the MAO, resulting in the improvement of cognitive function [40]. Moreover, Luteolin-7-O-glucoside, an important constituent in TA extract, exhibits potent MAO inhibitory potential [36]. A clear observation was made that Q-LNP successfully achieved the normalization of DA, 5-HT and MAO. The probable cause of this phenomenon might be attributed to a variety of scenarios, such as increased bioavailability and permeability [1].

Inflammation is frequently linked to diabetes problems, including diabetic cognitive impairments and OS [2]. Most neuropathological diseases show an elevation in proinflammatory cytokines levels. For example, diabetes induces the production of TNF-α and IL-1β which are known as the major proinflammatory cytokines that result in diabetes-associated cognitive decline [2], and TNF-α can be generated by hyperglycemia and enhance the transcription factor NF-κB via TNF-α receptor activation on the surface of neurons and glia cells [41]. Cytokines might potentially stimulate NO synthesis, which is mediated by iNOS [42]. Results of the current study showed considerably higher levels of TNF-α and NF-kB levels, as well as IL-1β and iNOS gene expression in the cerebral cortex of diabetic rats. These findings are consistent with others who showed increased levels of TNF-α, NF-κB, IL-1β, and iNOS in the brains of diabetic rats [2,41,42]. However, oral administration of Q-LNP, BCA, and TA extract restored these levels. Earlier studies also reported that Q treatment lowers the production of proinflammatory cytokines [2,41]. Furthermore, BCA has been found to protect neuron damage by inhibiting the production of proinflammatory cytokines such as TNF-α, IL-1β, and iNOS, which leads to NF-κB suppression [43]. Others reported TA extract plays a key role in decreasing the generation of proinflammatory cytokines associated with diabetic complications [8].

Hyperglycemia has been shown as a risk factor for Alzheimer’s disease [44]. Several studies reported that hyperglycemia promotes amyloid beta deposition on brain lesions, impairs neuroinflammation, and compromises neuronal integrity, all of which contribute to neurodegeneration [45]. The current diabetes model was linked to elevated APP and BACE expression as well as reduced PSEN2 expression. These results are in agreement with those of others. Khalaf et al. [46] showed an increase in APP and BACE brain expression in diabetic rats. Amyloid beta is formed via the cleavage of APP by β- and γ-secretases. Tau hyperphosphorylation promotes aggregation, resulting in neurofibrillary tangles [45]. Furthermore, elevated BACE expression allowed APP processing via the β-secretase pathway rather than the α-secretase pathway, resulting in greater Aβ production [47]. PSEN2 is the most common γ-secretase that modulates the release of proinflammatory cytokines that function as a neuroprotective marker [45]. PSEN2 expression was shown to be suppressed in AlCl3-induced AD [48]. Conferring to our findings, treating diabetic rats with Q-LNP, BCA, and TA extract generated a substantial reduction in APP and BACE brain expression while increasing PSEN2 expression. It is widely known that Q has anti-amyloidogenic properties by inhibiting amyloid aggregation via proteasome activation [5], and it modulates APP processing and increases Aβ clearance [49,50]. Other studies have shown that Q inhibits BACE enzyme activity [10]. Paris et al. [51] documented that NF-κB regulates Aβ synthesis by regulating APP cleavage and that Q-induced NF-κB suppression impacts BACE expression regulation. Additionally, BCA has been shown to have neuroprotective properties by inhibiting BACE activity [52].

Insulin action is normally triggered when it adheres to its receptor, resulting in auto-phosphorylation. This then stimulates the PI3K/Akt pathway, resulting in GLUT 4 translocation to the cell membrane and increased glucose uptake [53]. Impaired insulin signaling increases the chance of AD [3]. Our findings reveal that during diabetes, IR, PI3K, AKT, AMPK, and FOXO-1 gene expression in the brain are dramatically downregulated, but PPAR-γ gene expression is significantly increased, leading to hyperglycemic condition in diabetic rat. These results are consistent with those of Xavier et al. [54], who reported considerable downregulation of IR gene expression in the skeletal muscle of STZ-induced diabetic rats. Moreover, STZ-treated rats had lower PI3K/Akt/mTOR signaling in the brain, indicating that STZ may impede signal transduction via Akt in addition to interfering with hypothalamic transmission [5]. It has also been established that diabetes is related with decreased AMPK activation as well as impaired insulin signaling [55]. In our results, the near absence of AMPK expression in the diabetic group is likely to have a number of negative consequences for metabolism. One possible explanation for this finding is that hyperglycemia and fatty acids in diabetes can damage AMPK and impair its function. Another possibility is that inflammation, which is also common in diabetes, can suppress AMPK expression [55,56]. As for diabetes; it also increased the expression of iNOS, NF-κB, and PPAR-γ but decreased that of FOXO-1 in the cortical tissue, supporting the importance of the inflammatory process in neuronal death [5]. These findings suggest that insulin signaling dysregulation via the PI3K/Akt pathway is a crucial component in the pathophysiology of hyperglycemia and neurodegeneration found in T2DM. In contrast, treatment of diabetic rats with Q-LNP, BCA, and TA extract were able to increase the gene expression of IR, PI3K, AKT, AMPK, and FOXO-1 while suppressing PPAR-γ gene expression in the brain. These results are consistent with those of Dhanya et al. [56] who reported that Q administration increased mRNA levels of AMPK and AKT. It also inhibits the expression of PPAR-γ, implying its capacity to reduce body weight and fat mass [57]. Further, BCA was able to alleviate the decrease in PI3K/Akt/mTOR signaling, resulting in neuroprotective consequences [58].

GSK-3β is a vital kinase in insulin signaling transduction and tau phosphorylation. One of the key characteristics of insulin resistance is impaired signal transduction of the insulin/PI3K/Akt signaling pathway, which leads to an increase in GSK-3β [4]. The current diabetes model was associated with significant elevation of GSK-3β, which follows the findings displayed by Gomaa et al. [59] reporting that diabetic rats displayed impaired cognitive performance function and elevated GSK-3β levels. Nevertheless, treatment of diabetic rats with Q-LNP, BCA, and TA extract reduced GSK-3β hyperactivity in the cortical tissue. It has been demonstrated that Q has a neuroprotective effect by inhibiting GSK-3β [60]. Furthermore, reports exposed that isoflavones prevent neuronal damage via the inhibition of GSK-3β [61], and several studies have reported the antidiabetic effects of plant extract administration trough the reduction in GSK-3β level [62].

In the current research, diabetes induced multifocal histological changes. These were in agreement with numerous previous studies that described similar structural changes in the cerebral cortex have been reported by others [63,64]. Treatment of diabetic rats with Q-LNP, BCA, and TA extract, on the other hand, resulted in a slight improvement in neuronal cells. These findings are in the same line as those represented by Ebrahimpour et al. [26], who found that Q alleviated abnormal histological signs in diabetic brains. Moreover, the effectiveness of isoflavones for the prevention and treatment of T2DM and related complications has been assessed in several studies.

Regarding GFAP immunohistochemical staining, our results revealed the occurrence of neuroinflammation in T2DM [65] owing to significant immunoreactivity reaction in the cerebral cortex segment of the diabetic group. Q-LNP, BCA, and TA extract administration decreased the number and intensity of GFAP positive cells in diabetic rats. The neuroprotective mechanisms of these treatments are unknown, but they may involve regulation of the transcription factor NF-κB, an essential downstream target of the PI3K/AKT signaling pathway that regulates inflammatory gene expression [2,41,43].

## 5. Conclusion

Our results indicate that Q-LNP, BCA, and TA act as neurotherapeutic agents that protest against diabetic-induced neurotoxicity. These treatments possess anti-diabetic, antioxidative, and anti-inflammatory capabilities via modulation of the PI3K/Akt/ GSK-3β signaling pathway. The current study is novel in its approach to this topic by shedding light on the synergistic effects of TA bioflavonoids in attenuating hyperglycemia-induced nervous system impairments. The findings of this study suggest that TA bioflavonoids have a promising role in the treatment and management of diabetes-induced cerebral cortical damage. Further research is needed to validate these findings in clinical trials and to determine the optimal dosage and formulation for human use.

## Supporting information

S1 Graphical abstract (PDF)

S1 Raw images (PDF)
