# Supplementary material for: Neurotherapeutic effects of quercetin-loaded nanoparticles and Biochanin-A extracted from Trifolium alexandrinum on PI3K/Akt/GSK-3β signaling in the cerebral cortex of male diabetic rats
Source: PLoS One. 2024 Apr 29;19(4):e0301355. doi: 10.1371/journal.pone.0301355 (PMC11057738; doi:10.1371/journal.pone.0301355)
Supplement: S1 Graphical abstract — (PDF) [file pone.0301355.s001.pdf]

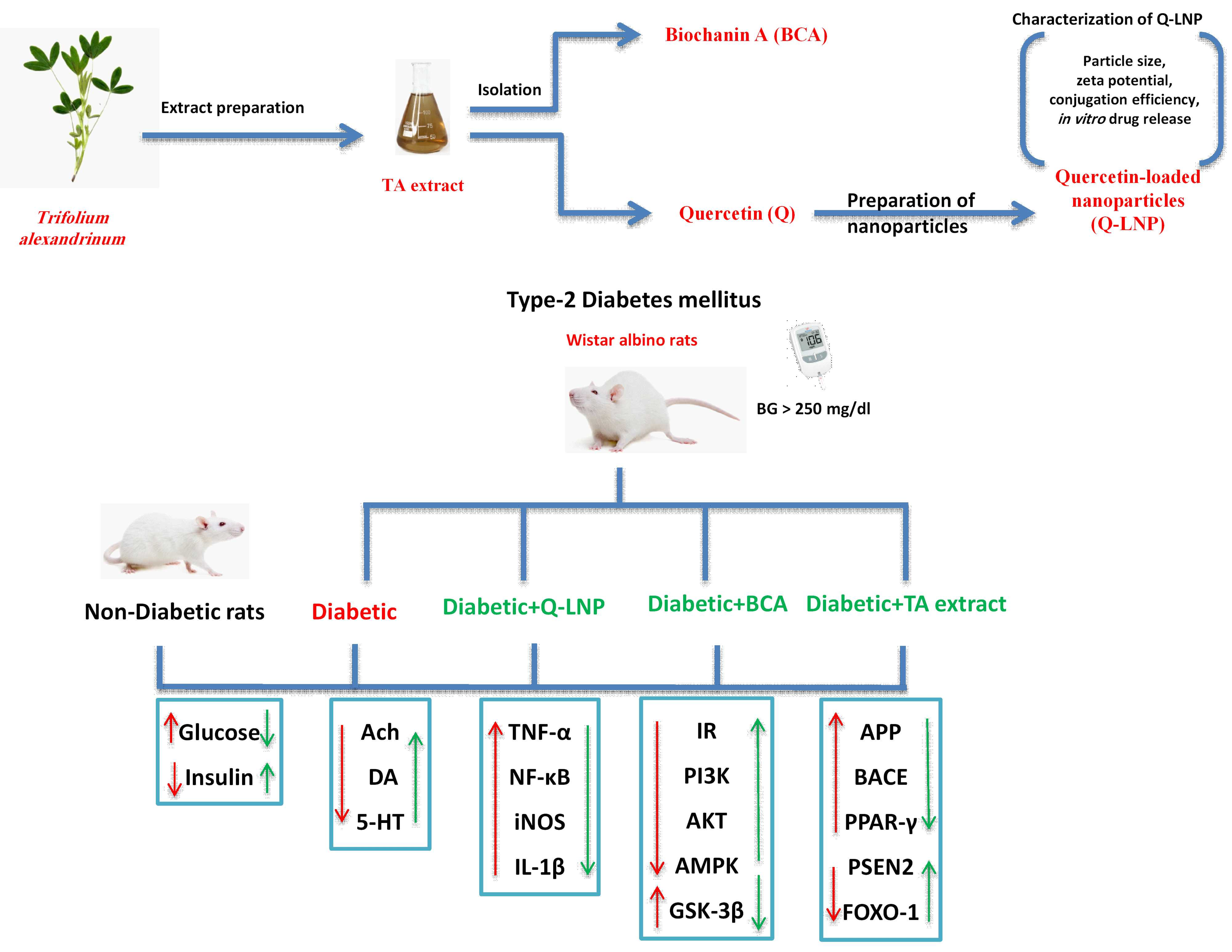
Trifolium alexandrinum
Extract preparation
TA extract
Isolation
Biochanin A (BCA)
Quercetin (Q)
Preparation of nanoparticles
Quercetin-loaded nanoparticles (Q-LNP)
Characterization of Q-LNP
Particle size, zeta potential, conjugation efficiency, in vitro drug release
Type-2 Diabetes mellitus
Wistar albino rats
BG > 250 mg/dl
Non-Diabetic rats
Diabetic
Diabetic+Q-LNP
Diabetic+BCA
Diabetic+TA extract
Glucose
Insulin
Ach
DA
5-HT
TNF-α
NF-κB
iNOS
IL-1β
IR
PI3K
AKT
AMPK
GSK-3β
APP
BACE
PPAR-γ
PSEN2
FOXO-1
