# Supplementary material for: Neurotherapeutic effects of quercetin-loaded nanoparticles and Biochanin-A extracted from Trifolium alexandrinum on PI3K/Akt/GSK-3β signaling in the cerebral cortex of male diabetic rats
Source: PLoS One. 2024 Apr 29;19(4):e0301355. doi: 10.1371/journal.pone.0301355 (PMC11057738; doi:10.1371/journal.pone.0301355)
Supplement: S1 Raw images — (PDF) [file pone.0301355.s002.pdf]

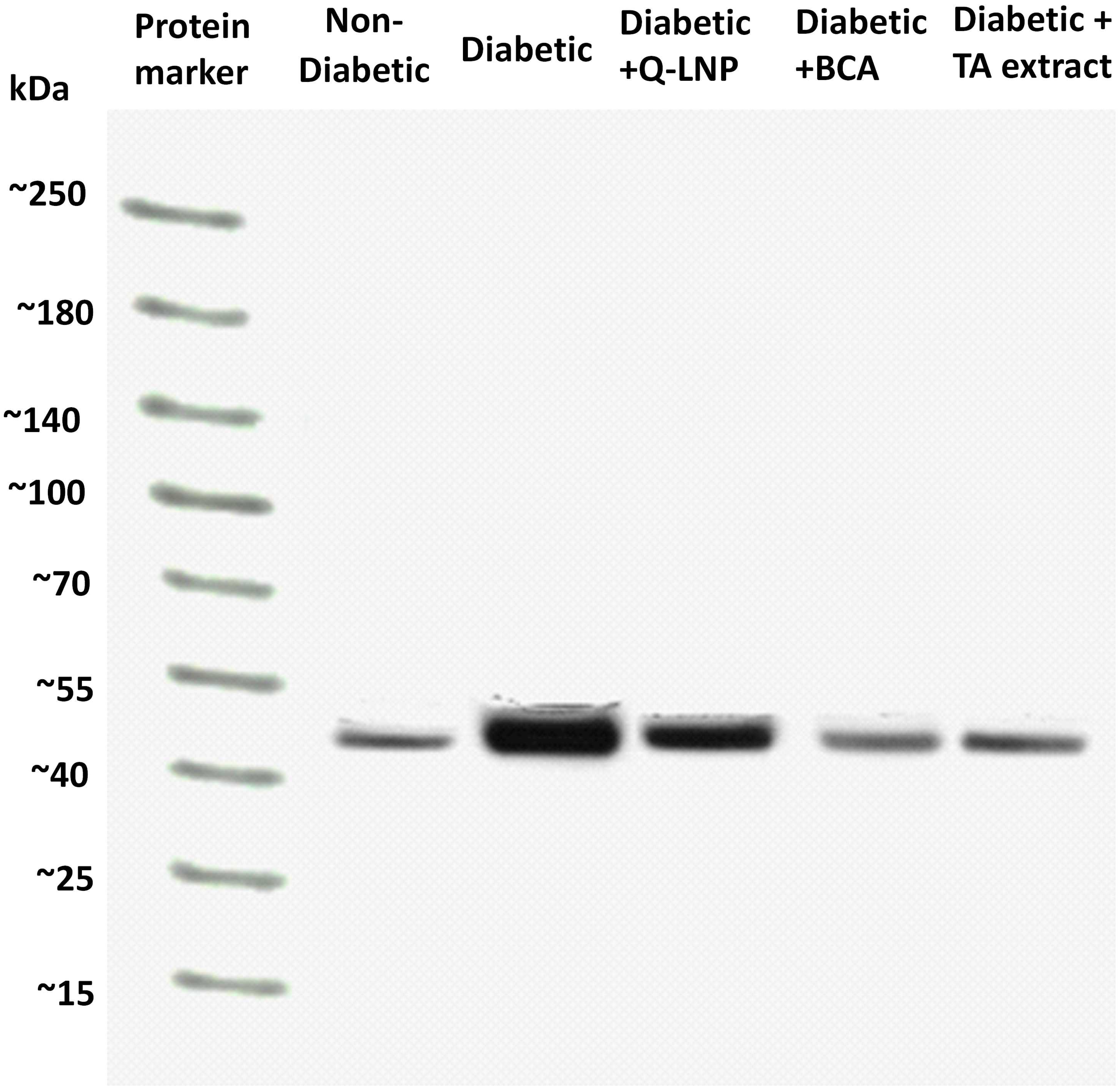

20 µg protein concentrations from each sample was loaded from left to right.

A CCD camera-based imager was used to collect the chemiluminescent signals.

Figure 4 was generated from this image

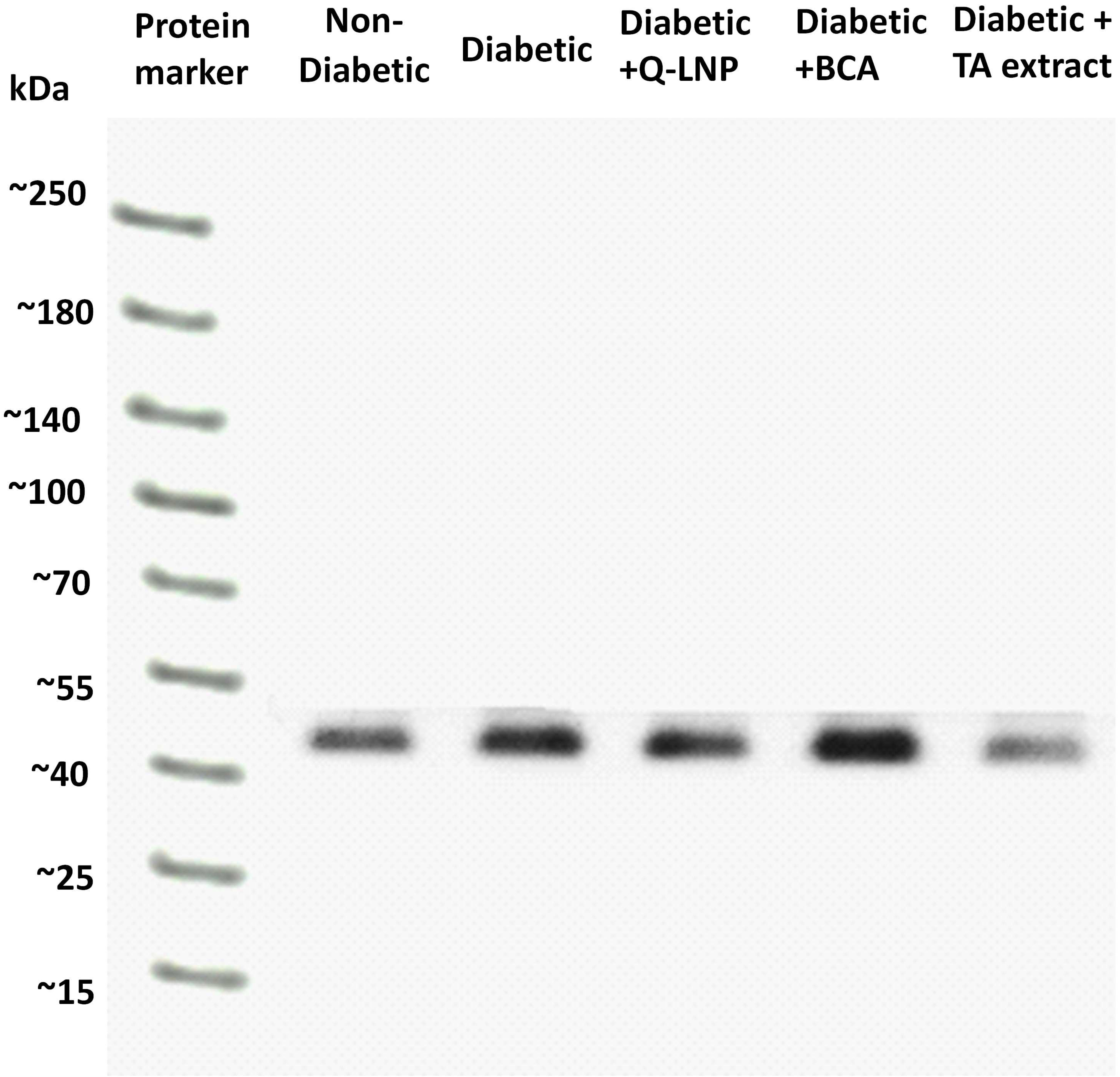

20 µg protein concentrations from each sample was loaded from left to right.

A CCD camera-based imager was used to collect the chemiluminescent signals.

Figure 4 was generated from this image
